# Incidence of Vancomycin-Resistant *Staphylococcus aureus* Strains among Patients with Urinary Tract Infections

**DOI:** 10.3390/antibiotics11030408

**Published:** 2022-03-18

**Authors:** Samy Selim, Osama Ahmed Faried, Mohammed S. Almuhayawi, Fayez M. Saleh, Mohamed Sharaf, Nihal El Nahhas, Mona Warrad

**Affiliations:** 1 Department of Clinical Laboratory Sciences, College of Applied Medical Sciences, Jouf University, Sakaka 72341, Saudi Arabia; 2 Medical Microbiology and Immunology Department, Faculty of Medicine, Beni-Suef University, Beni-Suef 62513, Egypt; sadomm2008@gmail.com; 3 Department of Medical Microbiology and Parasitology, Faculty of Medicine, King Abdulaziz University, Jeddah 21589, Saudi Arabia; msalmuhayawi@kau.edu.sa; 4 Department of Medical Microbiology, Faculty of Medicine, University of Tabuk, Tabuk 71491, Saudi Arabia; fsaleh@ut.edu.sa; 5 Department of Biochemistry, Faculty of Agriculture, AL-Azhar University, Cairo 11651, Egypt; mohamedkamel@azhar.edu.eg; 6 Department of Biochemistry and Molecular Biology, College of Marine Life Sciences, Ocean University of China, Qingdao 266003, China; 7 Department of Botany and Microbiology, Faculty of Science, Alexandria University, Alexandria 21526, Egypt; nihal.elnahhas@alexu.edu.eg; 8 Department of Clinical Laboratory Sciences, College of Applied Medical Sciences at Al-Quriat, Jouf University, Al-Quriat 77454, Saudi Arabia; mfwarad@ju.edu.sa

**Keywords:** plasmid, genotyping, vancomycin resistance, *Staphylococcus aureus*, VRSA, urinary tract infections, Saudi Arabia

## Abstract

There has been a substantial rise in the number of vancomycin-resistant *Staphylococcus aureus* (VRSA) strains during the last several years. The proportion of vancomycin-resistant strains among isolated *S. aureus* has risen steadily in recent years, with the first spike occurring in critical care units and thereafter in general hospital wards. *S. aureus* isolates from urinary tract infection patients were studied for their prevalence and antibiotic resistance. From 292 urine samples, 103 bacterial strains (35.3%) were identified as *S. aureus*. Various antibiotics were used to test the isolates’ antibacterial resistance profiles. Antibiotic resistance to erythromycin was found in most bacterial isolates, whereas tobramycin antibiotic sensitivity was found in most of them. Vancomycin resistance was found in 23 of all *S. aureus* isolates in this study. Analysis for β-lactamase found that 71% of *S. aureus* isolates were positive in all isolates. There was a single plasmid with a molecular weight of 39.306 Kbp in five selected VRSA isolates that was subjected to plasmid analysis. There was evidence of vancomycin resistance among the *S. aureus* isolates collected from UTI patients in this investigation. This vancomycin resistance pretenses a challenge in the treatment of *S. aureus* infections and the need to precisely recognize persons who require last-resort medication such as tobramycin.

## 1. Introduction

Urinary tract infections (UTIs) are frequent human microbial illnesses that affect the urinary tract—the kidneys, bladder, urethra, and prostate [1]. This disease has a direct and indirect impact on people’s lives, and it is a global problem. Furthermore, these disorders are becoming a leading source of morbidity [2]. UTIs are predicted to affect 150 million individuals worldwide each year [3]. The cost of healthcare in the United States is estimated to be $6 billion [4]. UTIs are the second most common cause of human illness [5]. UTIs are responsible for more than 1 million emergency room visits and 100,000 hospitalizations in the United States each year [4]. UTIs can be classified as either community- or nosocomial acquired [3]. A community-acquired urinary tract infection (CA-UTI), which occurs in the community after less than 48 h of hospitalization, is one of the two types of UTIs. Nosocomially acquired urinary tract infections (N-UTI) emerge 48 h after hospital admission or three days after release [6]. UTI prevalence is influenced by characteristics such as age, gender, catheterization, inpatient treatment, and long-term use of antimicrobials [3]. The majority of UTIs are caused by Gram-negative bacteria, which account for 90%, but only 10% of bacterial infections [7]. *Escherichia coli* is the most prevalent cause of UTIs, accounting for 65–90% of infections [8,9]. *Enterococcus* species, *Klebsiella pneumoniae*, *Citrobacter* species, *Pseudomonas aeruginosa*, and coagulase-negative *staphylococci* (CoNS) are among the other uropathogens that cause UTIs [10]. In general, urinary tract infections caused by *S. aureus* are rather rare. However, in certain individuals, *S. aureus* produces ascending urinary tract colonization and infection, which is commonly subsequent to staphylococcal bacteremia originating elsewhere (e.g., in instances of endocarditis), and indwelling catheters and other urinary tract instruments increase the risk of *S. aureus* transmission [11,12].

*Staphylococcus aureus* has flourished in humans because its capacity to adapt its ability to acquire antibiotic resistance has made it an important pathogen in a wide range of settings [13,14]. More than half of all hospital-acquired *S aureus* infections are caused by methicillin-resistant *S. aureus* (MRSA) [15]. Antibiotic resistance in *S. aureus* has been linked to the plasmid-mediated synthesis of β-lactamases and other activities [16,17,18].

Until the 1970s, vancomycin was the antibiotic of choice for treating infections caused by MRSA, but increased usage of vancomycin has led to the development of two forms of glycopeptide-resistant *S. aureus* [19]. One of the two strains, vancomycin-intermediate *S. aureus* (VISA), is associated with a thicker and weakly cross-linked cell wall, which results in the buildup of targets at the cell periphery that sequester glycopeptides [20,21]. There is a second form, vancomycin-resistant *S. aureus* (VRSA), which results in high-level resistance [22,23]. Because they were identified in many nations across the world, these currently rare strains are extremely important [24,25]. Evidence is accumulating that they will become more frequent over time, as the number of vancomycin-resistant infections reported grows year after year [26,27]. *S. aureus* heteroresistance to vancomycin has been observed, and this might lead to the development of more resistant strains over time. According to several research studies, the prevalence rates of VRSA range from as low as 1.3 % to as high as 20% [24,28]. Resistance was predominantly seen in patients from intensive care units, followed by medical wards, before surgery [28]. Patients with bacteremia and/or endocarditis are at greater risk of dying from VRSA infections, making it imperative that a treatment regimen with high bactericidal effectiveness be considered when making treatment decisions [29,30,31,32]. There has always been worry about the evolution of vancomycin resistance in MRSA isolates via plasmid transfer [33,34]. VRSA is often regarded as the most serious threat to patients due to *S. aureus*’ aggressive characteristics, despite the fact that vancomycin resistance in this bacterium is still relatively rare in humans [19]. Due to *S. aureus*’ aggressive nature and the limited treatment options for MRSA infections, the development of VRSA poses a substantial danger to public health [35]. Furthermore, the possibility of these microorganisms being spread from one patient to another is also worrisome [36,37]. The purpose of this study was to explore the prevalence of VRSA isolated from patients with UTIs.

## 2. Materials and Methods

This cross-sectional descriptive study was carried out during the period from October 2020 to February 2022. A total of 103 different MRSA *S. aureus* strains were isolated from 292 urine samples of patients attending the outpatient clinics in the Prince Mutaib Bin Abdulaziz Hospital in Sakaka, Al Jouf, Saudi Arabia. Bacterial isolates were handled in the microbiology laboratory, the Department of Clinical Laboratory Sciences, the College of Applied Medical Science, Jouf University, Saudi Arabia.

### 2.1. Bacterial Isolates

Specimens were incubated at 37 °C for 24 h on Blood Agar and Mac-Conkey Agar. Staphylococcal isolates were identified using biochemical and morphological approaches. Multiple biochemical tests for the confirmation of *S. aureus* were performed on the Gram-positive cocci in clusters detected under a microscope. We identified *S. aureus* based on the presence of catalase and oxidase as well as coagulase activity and DNase activity in the *S. aureus* colonies on mannitol salt agar. The results of these biochemical tests were used to identify the organism. Presumptive VRSA confirmed by a Vitek 2 identification card (bioMerieux, Marcy l’Etoile, France) was used for automated strain identification according to the manufacturer’s instructions [37,38].

### 2.2. Antibiotic Susceptibility Testing

The original stock cultures of *S. aureus* were used in all tests to avoid the loss of antibiotic resistance that might occur when frequently subculturing. From the pure and fresh *S. aureus* growth, 0.5 McFarland suspensions were prepared using sterile normal saline [39]. Then, the suspension was inoculated on Mueller–Hinton agar (MHA) for modified Kirby–Bauer disk-diffusion susceptibility analysis. Discs of antibiotics (imipenem (10 µg), oflaxacin (5 µg), amoxicillin (10 µg), erythromycin (30 µg), amikacin (30 µg), tobramycin (30 µg), and vancomycin (5 µg)) were placed on the agar surface after the inoculum had dried. After 24 h of incubation at 37 °C, the diameter in mm of clear zones around the antibiotic discs suggesting bacterial growth inhibition was evaluated. For vancomycin resistance confirmation, the breakpoints of minimum inhibitory concentrations (MIC) were measured for strains displaying resistance in disk-diffusion susceptibility analysis. The vancomycin broth MIC ≤ 2 μg/mL is considered sensitive, 2–4 µg/mL MIC is taken as intermediate, and vancomycin broth MIC ≥ 16 μg/mL is considered resistant to vancomycin. *S. aureus* ATCC 29213 MIC of vancomycin broth with a value of 0.5–2.0 µg/mL was used as a control strain to measure the performance of vancomycin.

### 2.3. β-Lactamase Detection

This test was carried out as explained by Odugbemi et al. [40]. Seven-centimeter-long strips of starch paper were cut and sterilized in 70% ethanol. Following this, the strips were soaked for 10 min each in a solution of benzylpenicillin (1000 units) in phosphate buffer. Sterile Petri dishes were used to distribute them. The test paper was then infected with cultures of tested (18 to 24 h old) organisms grown on nutrient agar and dispersed across an area of 2 to 3 mm. It was then inundated with Gram iodine solution after 30 min of incubation at 37 °C in the Petri dishes. Within 30 s of applying this, the starch paper was completely black. β-lactamase production was shown by colonies with decolorized zones thereafter. 

### 2.4. Plasmid Studies

Mini-prep alkaline extraction was used to isolate the plasmid [41]. The tris-borate EDTA (TBE) buffer was used for agarose gel electrophoresis. We added 1% agarose (stock solution of 10 mg/mL) to the TBE buffer and 5 µL of ethidium bromide (stock solution of 10 µg/mL) to make gels. The agarose gel was run for two hours at 90 mV with 100 µL of MgCl_2_ (100 mM), 10 mM EDTA, and a loading buffer (5 µL). For this experiment, we used the same gel to run both the marker plasmid and a reference DNA sample. We then used *BamH I*, *EcoR I*, and *Hind III* (Roche Diagnostics GmbH in Mannheim, Germany) endonuclease enzymes for the digestion of plasmid, as well as 1 µL of the high salt digestion buffer (1 M NaCl and 500 mM Tris-HCl), 2 µL of deionized water, 1 µL of the pure DNA sample, and 2 µL of an endonuclease enzymes digestion buffer (pH 7.5).

## 3. Results

**Figure 1:**
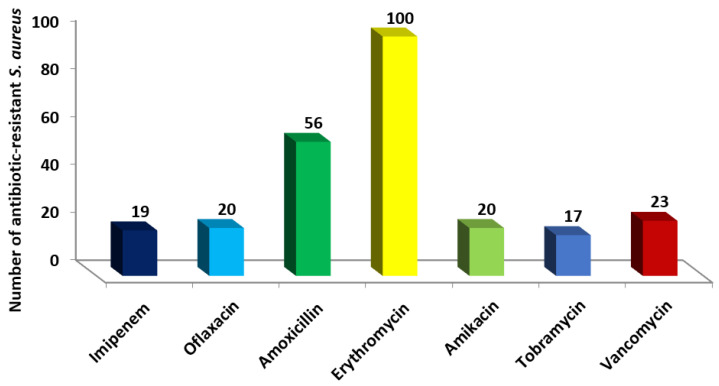
Number of antibiotic-resistant *S. aureus* isolates obtained from urine samples against studied antimicrobial agents.

There were 292 urine samples from patients admitted to the hospital that tested positive for the staphylococcal infection. A total of 103 *S. aureus* isolates were found in urine samples. The prevalence of *S. aureus* in urine samples was 35.3%. There are *S. aureus* isolates from urine samples whose antibiotic resistance profiles were given in Figure 1. Antibiotic resistance to erythromycin was found in most *S. aureus* isolates (97%), whereas tobramycin antibiotic sensitivity (16.5%) was found in most of them. Twenty-three *S. aureus* isolates (22.3%) were resistant to vancomycin (Figure 1). Testing for the synthesis of β-lactamase enzymes was carried out on all *S. aureus* strains. Among the *S. aureus* isolates studied, 71% were able to generate β-lactamase.

**Figure 2:**
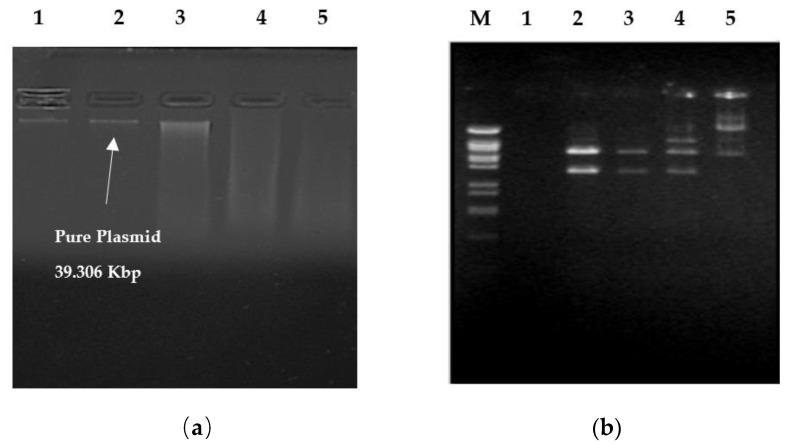
Agarose gel electrophoresis of plasmids obtained from (**a**) pure plasmids of VRSA isolates without cutting and (**b**) VRSA plasmid (Lanes 2–4) restriction arrays digested using the restriction enzymes *BamH I*, *EcoR I*, and *Hind III*, respectively. Lane M is the 100 bp ladder and lane 1 is deionized water.

Only one plasmid is present in five selected studied VRSA isolates of the 23 total VRSA (Figure 2a). A certain plasmid from one of the VRSA isolates was digested with *Hind III*, *EcoR I*, and *BamH I* as shown in Figure 2b. The number of recognition sites, number of fragments, and approximate molecular size of the plasmids are stated in Table 1. *S. aureus* plasmids were found to have a molecular weight of 39.306 Kbp.

## 4. Discussion

*S. aureus* is one of the most common Gram-positive bacteria implicated in urinary tract infections (UTIs). Increasing antibiotic resistance in such organisms is of concern to public health [42]. *S. aureus* strains have shown an alarming rise in resistance to methicillin and decreased sensitivity to vancomycin [43]. As part of this investigation, the susceptibility pattern of *S. aureus* isolated from UTI was analyzed. *S. aureus* isolates were most effectively treated with tobramycicn, according to the findings of our investigation. During the last several years, the prevalence of VRSA has skyrocketed. According to several studies, the proportion of vancomycin-resistant strains among isolated *S. aureus* has risen steadily over the last few years, with the initial spike occurring in critical care units and then spreading to the rest of the hospital. *S. aureus* lacks a uniform mechanism for vancomycin resistance, while different genotypes and subtypes of resistance are found among *S. aureus* [44].

According to the information shown above, VRSA isolates show resistance to routinely prescribed antibiotics. This is primarily due to the widespread use of antibiotics and the selective hospital environment. Multiple antibiotic-resistance organisms have drawn the attention of many healthcare practitioners, and antibiotic resistance is a challenge in the treatment of UTIs [45,46,47,48,49]. Broad-spectrum medicines are suitable in this group, adapted to local resistance patterns and the most likely causal factor in the specific patient [50].

A genetic alteration and subsequent selection processes by antibiotics are the most common causes of antibiotic-resistant microorganisms. Several publications have documented antibiotic resistance in *S. aureus* caused by plasmids [47,48]. For pathogenic bacteria, plasmid-linked resistance is still essential. An investigation on the plasmid-linked resistance patterns of five VRSA isolates was conducted. At least one identical plasmid band was found in each of these strains after plasmid isolation [36,48,51,52,53,54].

Antibiotic resistance can be reduced by treating hospital workers who are known carriers of multi-resistant *S. aureus* strains [55]. There are those who believe the practice of greater personal and environmental hygiene will play a significant role in cutting down on the risk of cross-infection [36]. In our view, the findings from this study will help doctors better treat and lower the risk of complications from these conditions. Additionally, these findings have substantial implications for hospital formulary decision makers in the areas of antibiotic use, infection control, and public health [11,15,45]. According to our findings, molecular biology investigations are needed to identify the genes responsible for antimicrobial resistance patterns in *S. aureus* isolates [51].

## 5. Limitation

Clinical and other diagnostic data could not be included in the laboratory logbook due to a lack of patient details. We could not compare the bacterial isolates and UTI types since the laboratory logbook did not specify the kind of UTIs.

## 6. Conclusions

Our findings show that VRSAs were present among UTIs. MRSA’s decreased resistance to vancomycin highlights the need of finding new treatment options. It is critical for doctors to know which patients should be given last-ditch treatments such as linezolid and tobramycin. Antimicrobial resistance must be monitored on a regular basis and may be decreased by devising and implementing treatments depending on species to avoid serious health problems. Focusing on urine as a possible reservoir for infection may be a useful technique for prophylaxis, according to our study.

## Figures and Tables

**Table 1:** The restriction configurations of plasmids isolated from vancomycin-resistant *S. aureus*.

Endonuclease Enzymes	*Hind III*	*EcoR I*	*BamH I*
**No. of Recognition Sites**	6	6	8
**No. of Fragments**	5	5	7
**Size of Fragments (Kbp)**	23.00–6.557–5.148–4.973–3.530	22.000–5.804–49.73–3.530–2.037	9.416–7.421–5.148–5.100–3.430–3.530–2.322
**Plasmid Size (Kbp)**	39.306 Kbp		

## Data Availability

The datasets used or analyzed during the current study are available from the corresponding author on reasonable request.
